# Strain diversity of plant‐associated *Lactiplantibacillus plantarum*

**DOI:** 10.1111/1751-7915.13871

**Published:** 2021-06-25

**Authors:** Annabelle O. Yu, Elissa A. Goldman, Jason T. Brooks, Benjamin L. Golomb, Irene S. Yim, Velitchka Gotcheva, Angel Angelov, Eun Bae Kim, Maria L. Marco

**Affiliations:** ^1^ Department of Food Science and Technology University of California, Davis Davis CA USA; ^2^ Department of Biotechnology University of Food Technologies Plovdiv Bulgaria; ^3^ Department of Applied Animal Science Kangwon National University Chuncheon Gangwon‐Do South Korea

## Abstract

*Lactiplantibacillus plantarum* (formerly *Lactobacillus plantarum*) is a lactic acid bacteria species found on plants that is essential for many plant food fermentations. In this study, we investigated the intraspecific phenotypic and genetic diversity of 13 *L. plantarum* strains isolated from different plant foods, including fermented olives and tomatoes, cactus fruit, teff injera, wheat boza and wheat sourdough starter. We found that strains from the same or similar plant food types frequently exhibited similar carbohydrate metabolism and stress tolerance responses. The isolates from acidic, brine‐containing ferments (olives and tomatoes) were more resistant to MRS adjusted to pH 3.5 or containing 4% w/v NaCl, than those recovered from grain fermentations. Strains from fermented olives grew robustly on raffinose as the sole carbon source and were better able to grow in the presence of ethanol (8% v/v or sequential exposure of 8% (v/v) and then 12% (v/v) ethanol) than most isolates from other plant types and the reference strain NCIMB8826R. Cell free culture supernatants from the olive‐associated strains were also more effective at inhibiting growth of an olive spoilage strain of *Saccharomyces cerevisiae*. Multi‐locus sequence typing and comparative genomics indicated that isolates from the same source tended to be genetically related. However, despite these similarities, other traits were highly variable between strains from the same plant source, including the capacity for biofilm formation and survival at pH 2 or 50°C. Genomic comparisons were unable to resolve strain differences, with the exception of the most phenotypically impaired and robust isolates, highlighting the importance of utilizing phenotypic studies to investigate differences between strains of *L. plantarum*. The findings show that *L. plantarum* is adapted for growth on specific plants or plant food types, but that intraspecific variation may be important for ecological fitness and strain coexistence within individual habitats.

## Graphical abstract

The phenotypic and genetic properties were examined for 13 *Lactiplantibacillus plantarum* (formerly *Lactobacillus plantarum*) strains from (fermented) plant sources. The findings broadly show that strains obtained from the same or similar plant environments tend to be more genetically related and share similar carbohydrate utilization and stress tolerance capacities. However, there were still significant differences between all strains, irrespective of their source, indicating that intraspecific variation ‐is important for ecological fitness of ‐*L. plantarum* within individual habitats.

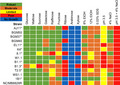

## Introduction

Certain lactic acid bacteria (LAB) required for food fermentations are recognized for their genetic and phenotypic diversity and have been classified as ‘nomadic’ or ‘generalist’ because of their broad habitat range (Duar *et al*., 2017; Choi *et al*., 2018; Yu *et al*., 2020). *Lactiplantibacillus plantarum* [formerly *Lactobacillus plantarum* (Zheng *et al*., 2020)] is included among those nomadic LAB (Duar *et al*., 2017) and is known for its significant intraspecific versatility (Molenaar *et al*., 2005; Siezen *et al*., 2010; Martino *et al*., 2016; Cen *et al*., 2020). *L. plantarum* is frequently isolated from fresh and fermented plant, meat and dairy foods and is an inhabitant of the gastrointestinal and vaginal tracts of humans and animals (Delgado *et al*., 2005; Aquilanti *et al*., 2007; Di Cagno *et al*., 2008; Yang *et al*., 2010; Ciocia *et al*., 2013; Jose *et al*., 2015; Zago *et al*., 2017; Parichehreh *et al*., 2018; Barache *et al*., 2020). This species is frequently required for or is involved in the production of numerous fermented foods (e.g. fermented olives, sauerkraut, salami, and sourdough), and certain strains are effective probiotics (Marco, 2010; Seddik *et al*., 2017; Crakes *et al*., 2019). Consistent with its host and environmental range, *L. plantarum* strains have larger genomes compared with LAB with narrow host ranges and also carry strain‐specific genes, often located on lifestyle adaptation islands (Molenaar *et al*., 2005; Sun *et al*., 2015; Zheng *et al*., 2015; Duar *et al*., 2017; Salvetti *et al*., 2018).

Despite the robust growth of *L. plantarum* in different host‐associated and food environments, *L. plantarum* genomes and cell properties have thus far shown limited correlations with isolation source across disparate habitats (Siezen *et al*., 2010; Martino *et al*., 2016). These findings indicate that either intraspecific variation of *L. plantarum* within individual sources is fortuitous and members of this species have not evolved for growth in specific habitats (Martino *et al*., 2016) or that this observed variation is the result of adaptive evolution of the *L. plantarum* species within certain habitats with the outcome of maximizing co‐occurrence by niche complementarity (Bolnick *et al*., 2011; Ehlers *et al*., 2016).

To begin to address these two hypotheses, we examined the intraspecies variation of a collection of *L. plantarum* strains isolated from fermented olives and other plant food types. *L. plantarum* is typically highly abundant in olive fermentations (Hurtado *et al*., 2012). Assessments of the population sizes of individual *L. plantarum* strains in olive fermentations over time have shown how these fermentations are highly dynamic, likely undergoing succession processes at both the species and strain levels (Zaragoza *et al*., 2017). These findings are notable because although LAB have received considerable attention for their contributions to plant fermentations, the diversity, abundance and importance of *L. plantarum* and other LAB in plant microbiomes are not well understood (Yu *et al*., 2020). It has been found that LAB in spontaneous (wild) plant food fermentations are subject to dispersal and selection constraints (Miller *et al*., 2019). However, adaptations expressed by these bacteria that are specific to plant environments and interactions between the same or highly related LAB species remain to be determined.

*Lactiplantibacillusplantarum* was isolated from olive fermentations (AJ11, BGM55, BGM37, BGM40 and EL11), tomato fermentations (T2.5 and WS1.1), teff injera fermentations (W1.1, B1.1 and B1.3), wheat sourdough starter (K4), wheat boza (8.1) and prickly pear cactus fruit (1B1) (Table 1). Some isolates were collected from the same source either at the same time (strain B1.1 and B1.3) or on different days over the course of fermentation (strains AJ11, BGM37 and BGM40). The strains were selected without considering special criteria or selective pressure. A reference strain from saliva (NCIMB8826R) was used for comparison. To investigate their phenotypic range, the *L. plantarum* strains were evaluated for growth on a variety of plant‐associated carbohydrates and during exposure to high NaCl [4% (v/v)], ethanol [8% and 12% (v/v)] or surfactant [sodium dodecyl sulfate (SDS, 0.03% (w/v)] stress. The isolates were measured for the capacity to grow at a low pH (pH 3.5) as well as survive (pH 2) and tolerate a high temperature (50°C) incubation. Biofilm formation and growth inhibition of *Saccharomyces cerevisiae* UCDFST 09‐448, a pectinolytic spoilage yeast (Golomb *et al*., 2013), were also tested. Lastly, to establish the genetic basis for the observed strain differences, multi‐locus sequence typing (MLST) and comparative genomics were performed.

**Table 1:** *Lactiplantibacillusplantarum* strains used in this study.

Strain name	Isolation source	Isolation date^a^	References
AJ11	Fermented olives; commercial fermentation	12/02/2010	Golomb *et al*. (2013)
BGM55	Fermented olives; pilot‐scale fermentation inoculated with *S. cerevisiae* 09‐448	03/07/2011	Golomb *et al*. (2013)
BGM37	Olive fermentation brine; commercial fermentation	01/04/2011	Golomb *et al*. (2013)
BGM40	Fermented olives; commercial fermentation	01/26/2011	Golomb *et al*. (2013)
EL11	Fermented olives; commercial fermentation	12/04/2009	Golomb *et al*. (2013)
K4	Wheat sourdough starter	09/15/2014	This study
8.1	Wheat boza	09/15/2014	This study
W1.1	White flour teff injera	04/04/2015	This study
B1.1	Brown flour teff injera	04/04/2015	This study
B1.3	Brown flour teff injera	04/04/2015	This study
T2.5	Fermented tomatoes	08/20/2015	This study
WS1.1	Fermented tomatoes (spoiled)	08/20/2015	This study
1B1	Ripe cactus fruit (*Opuntia ficus‐indicia*)	10/25/2011	Tyler *et al*. (2016)
NCIMB8826R	Human saliva	N/A	Yin *et al*. (2018)

^a^ Month/Day/Year. N/A, not available.

## Results

### Strain differentiation and phylogenetic analysis

The isolates were identified as *L. plantarum* by 16S rRNA gene sequence analysis and differentiated from the closely related species *Lactiplantibacillus pentosus* [formerly *Lactobacillus pentosus* (Zheng *et al*., 2020)] and *Lactiplantibacillus paraplantarum* [formerly *Lactobacillus* *paraplantarum* (Zheng *et al*., 2020)] by multiplex PCR targeting *recA* (Torriani *et al*., 2001).

**Fig. 1:**
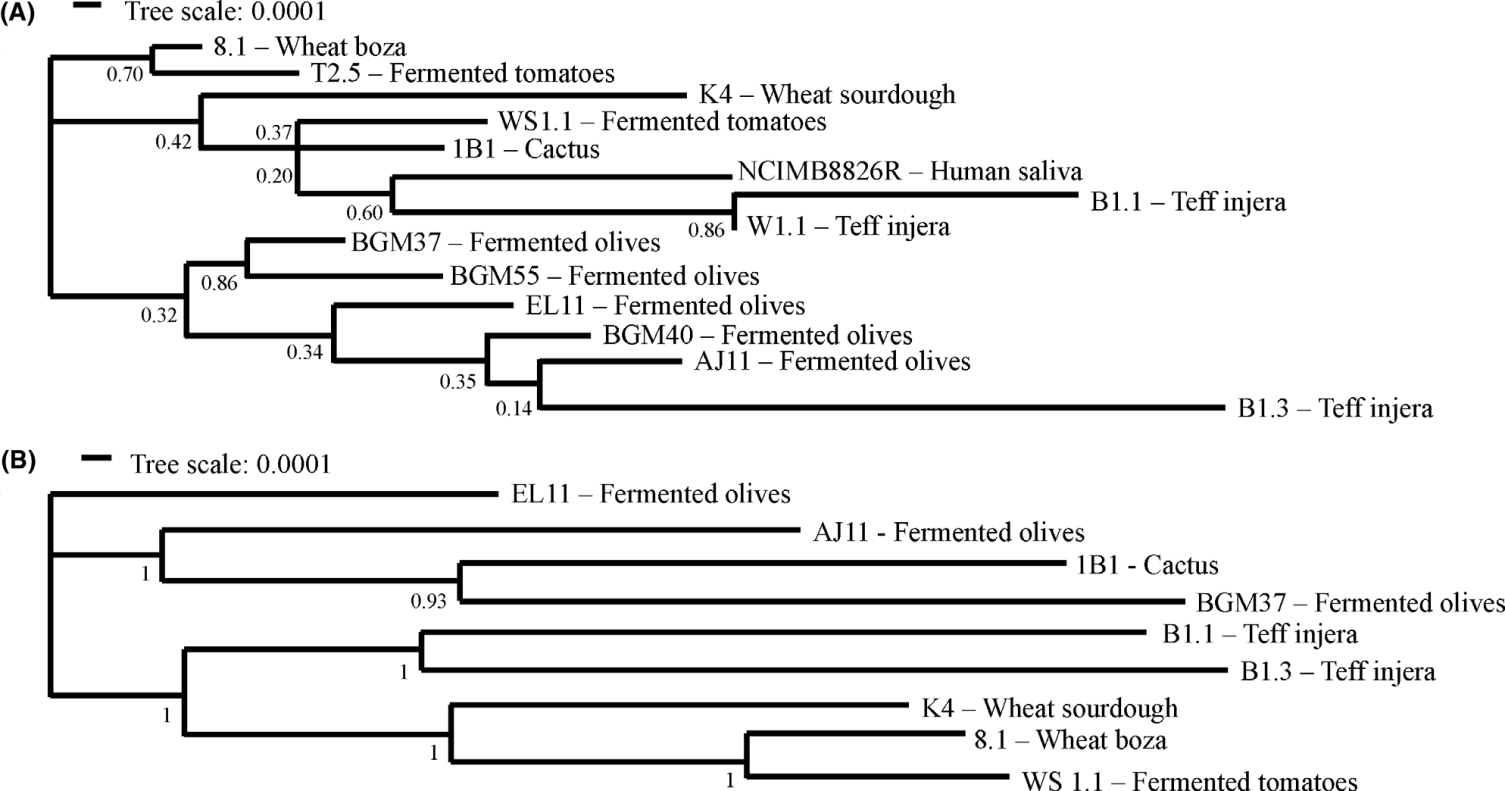
Phylogenetic relationships between *Lactiplantibacillus* *plantarum* strains. A. Phylogenetic relationships of 14 strains of *L. plantarum* based on MLST profiles with *pheS*, *pyrG*, *uvrC*, *recA*, *clpX*, *murC*, *groEL* and *murE* (Table S8). B. Relationships of nine *L. plantarum* strains based on concatenated core protein sequences using the maximum likelihood method with bootstrap values calculated from 500 replicates using Mega (7.0) (Kumar *et al*., 2016).

The strains were also found to have unique allelic multi‐locus sequence typing (MLST) sequence types (ST) (Table S1), thus confirming that they are genetically distinct and not derived from the same clonal populations. Among the eight genes tested by MLST, between 6 (*uvrC*) and 12 (*pyrG*) different alleles were found (Table S1). Phylogenetic analysis of the ST showed that the *L. plantarum* strains clustered into two clades (Fig. 1A). The isolates from fermented olives were contained in one clade, suggesting they are more closely related to each other and to the teff injera strain B1.3 than those retrieved from other sources. The two other strains from teff injera (B1.1 and W1.1) clustered together in the other clade which also contained NCIMB8826R and the strains isolated from wheat boza, sourdough, cactus fruit and fermented tomatoes (Fig. 1A). When examined in a MLST phylogenetic tree containing 264 other *L. plantarum* strains (Fig. S1), the *L. plantarum* isolates collected from fermented olives remained clustered closely together, whereas the others were distributed across the tree.

### Carbohydrate utilization capacities

**Fig. 2:**
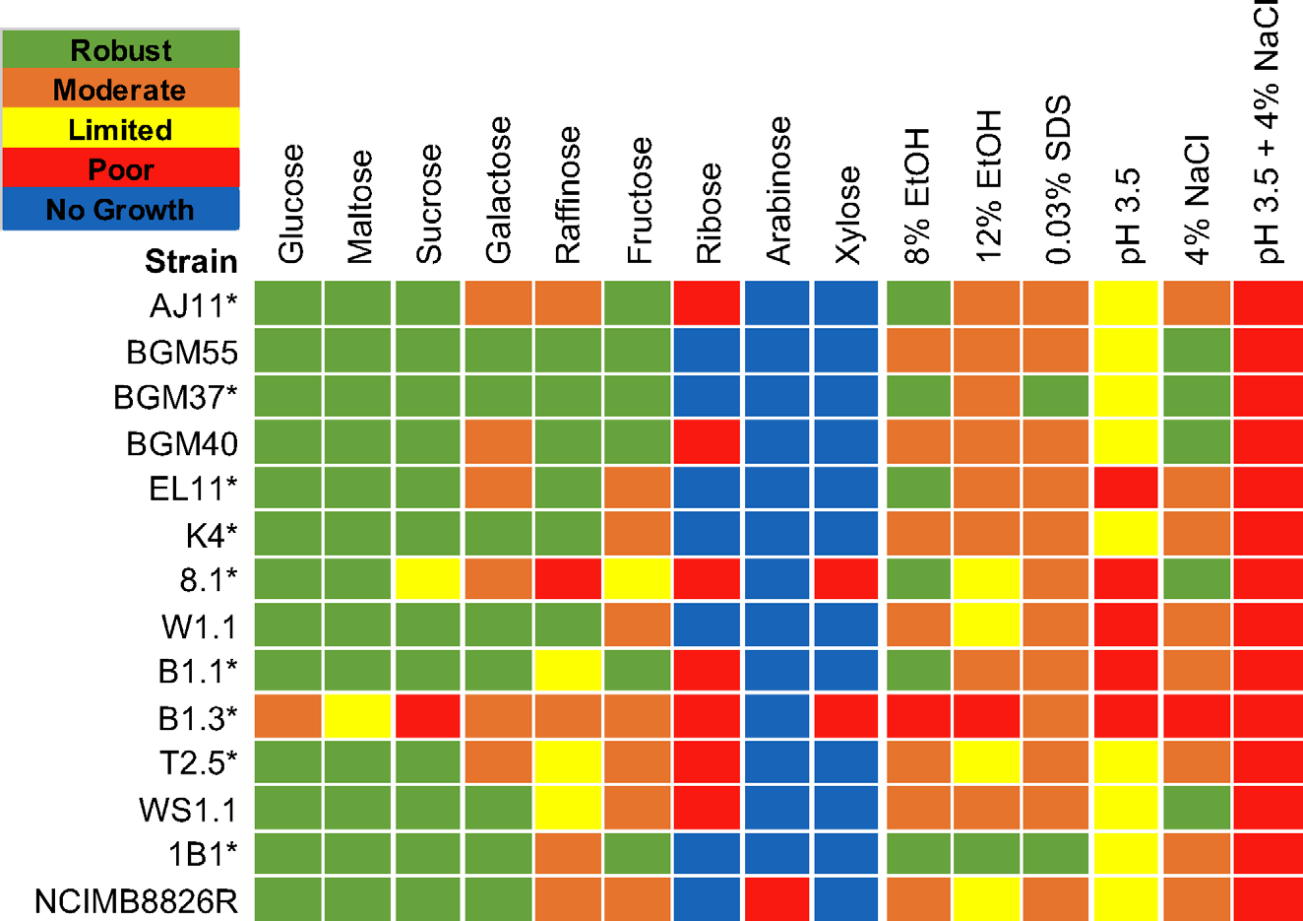
*Lactiplantibacillusplantarum* phenotype profiles. Area under the curve (AUC) values were used to illustrate *L. plantarum* capacities to grow in mMRS containing different sugars and in mMRS‐glucose in the presence of 8% (v/v) ethanol (EtOH), 8% (v/v) ethanol and then 12% (v/v) ethanol (12% ethanol), 0.03% (w/v) SDS, 4% (w/v) NaCl or set at pH 3.5 without or with 4% (w/v) NaCl. AUC values for the growth curves were ranked as ‘robust’ (AUC between 150 and 115), ‘moderate’ (AUC between 114 and 80), ‘limited’ (AUC between 79 and 45), ‘poor’ (AUC < 45) or ‘no growth’ (AUC was equivalent to the strain growth in mMRS lacking a carbohydrate source). *L. plantarum* growth in mMRS‐glucose supplemented with an equal volume of water instead of ethanol, NaCl or SDS was not significantly different compared to growth in mMRS‐glucose (*P* > 0.05). * indicates strains examined by whole genome sequencing.

**Fig. 3:**
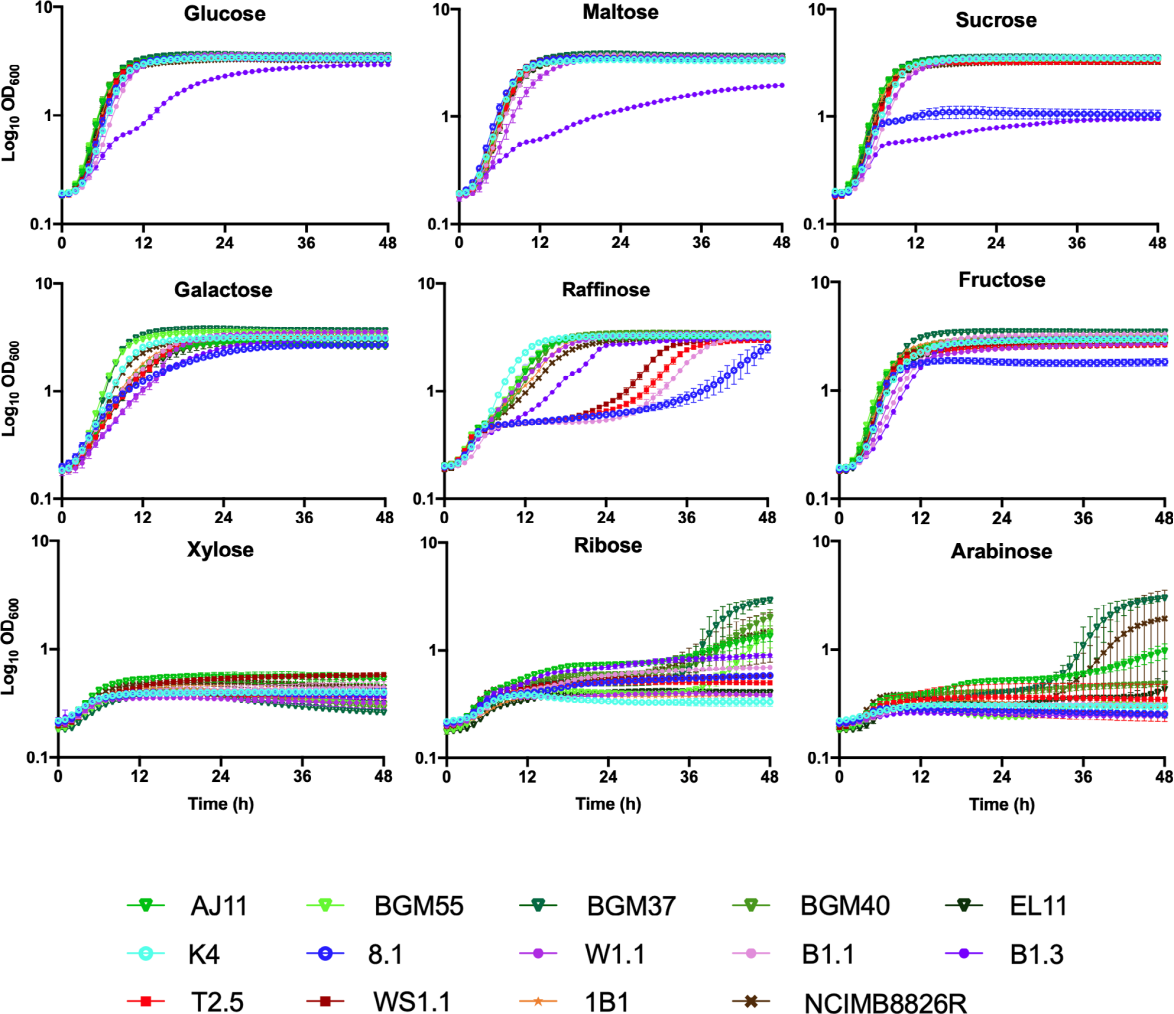
Growth of *Lactiplantibacillus* *plantarum* in mMRS containing different mono‐, di‐ and tri‐saccharides. *L. plantarum* was incubated in mMRS containing 2% (w/v) of each sugar at 30°C for 48 h. The avg ± stdev OD_600_ values of three replicates for each strain are shown.

The capacity of the *L. plantarum* strains to use different sugars for growth was measured using MRS, a complete medium commonly used for cultivation of LAB (De Man *et al*., 1960). To exclude metabolizable carbon sources, the MRS was modified (mMRS) to remove beef extract and dextrose. In mMRS containing glucose, maltose or sucrose, all *L. plantarum* strains except B1.3 (teff injera) and 8.1 (wheat boza) were found to have robust growth according to area under the curve (AUC) rankings (Figs 2 and 3; Table S2). Those strains which grew robustly reached maximum OD_600_ values within 12 h (Fig. 3; Table S3) and displayed growth rates ranging from a low of 0.31 ± 0.01 h^−1^ (strain W1.1 (teff injera) in maltose) to a high of 0.45 ± 0.01 h^−1^ [BGM37 (fermented olives) in glucose] (Table S4). By comparison, the growth rate of B1.3 was lower in glucose (0.20 ± 0.00 h^−1^) and maltose (0.15 ± 0.00 h^−1^) compared to the other strains (Fig. 3; Table S4). In mMRS‐sucrose, both B1.3 and 8.1 exhibited poor growth (Figs 2 and 3; Table S2).

All strains grew moderately to robustly when galactose was provided as the sole carbon source in mMRS (Figs 2 and 3; Table S2). Growth rates ranged from a low of 0.16 ± 0.003 h^−1^ [B1.3 (teff injera)] to a high of 0.42 ± 0.01 h^−1^ [BGM37 (fermented olives)] (Table S4). Final OD_600_ values measured after 24 h incubation ranged from 2.58 ± 0.05 [BGM40 (fermented olives)] to 3.62 ± 0.03 (BGM37) (Table S3). Because incubation in glucose‐containing MRS prior to exposure to mMRS‐galactose might result in carbon catabolite repression (Kremling *et al*., 2015), several strains with only moderate growth in that culture medium [AJ11, BGM40 and EL11 (fermented olives), 8.1 (wheat boza), B1.3 (teff injera) and T2.5 (fermented tomatoes)] were inoculated in succession into mMRS‐galactose. However, prior exposure to mMRS‐galactose did not result in higher AUC values (data not shown).

In mMRS with raffinose, all five *L. plantarum* strains isolated from fermented olives (BGM37, BGM55, BGM40, AJ11 and EL11) exhibited either moderate or robust growth (Figs 2 and 3; Tables S2–S4). Although strain W1.1 (teff injera) also grew robustly, the other strains isolated from teff and wheat fermentations (8.1, B1.1 and B1.3) and both strains isolated from fermented tomatoes (T2.5 and WS1.1) displayed limited or poor growth (Figs 2 and 3; Tables S2–S4). To address whether the poor growth of those isolates was due to carbon catabolite repression, serial passage in mMRS‐raffinose was performed. Notably, growth of four out of the five strains (B1.1, 8.1, T2.5 and WS1.1) was improved by successive cultivation in mMRS‐raffinose (Fig. S2).

When fructose was provided, all *L. plantarum* isolates except for strain 8.1 (wheat boza) exhibited either moderate or robust growth (Figs 2 and 3; Table S2). Similar to incubation in glucose and galactose, strain BGM37 (fermented olives) reached the highest OD_600_ (OD_600_ = 3.44 ± 0.03) (Table S3). Notably, growth of B1.3 (teff injera) was improved in mMRS‐fructose compared to the other sugars tested, as demonstrated by a higher growth rate (Table S4) and final OD_600_ (Table S3). Similar to the lack of effect on AUC values found after successive passage in the presence of mMRS‐galactose, no significant differences in growth were found for any of the 14 strains after multiple passages in mMRS‐fructose (data not shown).

Growth of *L. plantarum* was poor in mMRS containing xylose, ribose or arabinose. Only four olive‐associated strains (AJ11, BGM55, BGM37 and BGM40) and NCIMB8826R grew in the presence of mMRS‐ribose or mMRS‐arabinose and none grew in mMRS‐xylose (Figs 2 and 3; Tables S2–S4). After 38 h in mMRS‐ribose, the OD_600_ values for those strains ranged from a low of 1.36 ± 0.15 [AJ11 (fermented olives)] to a high of 2.93 ± 0.17 [BGM37 (fermented olives)] (Fig. 3; Table S3). In mMRS with arabinose, only NCIMB8826R and BGM37 grew, reaching an OD_600_ of 1.94 ± 1.56 and 2.99 ± 0.14 respectively (Fig. 3; Table S3). To investigate whether growth could be improved by prior exposure to those pentose sugars, strains AJ11, BGM37, 8.1 and NCIMB8826R were incubated with successive passages in mMRS‐ribose or mMRS‐arabinose. This resulted in shorter lag phase times and higher final OD_600_ values for AJ11, BGM37 and NCIMB8826R in both media (Figs S3 and S4). By comparison, no difference in growth was observed for strain 8.1 (wheat boza) in mMRS‐ribose or mMRS‐arabinose irrespective of the adaptation period (Figs S3 and S4).

### Growth in the presence of ethanol

**Fig. 4:**
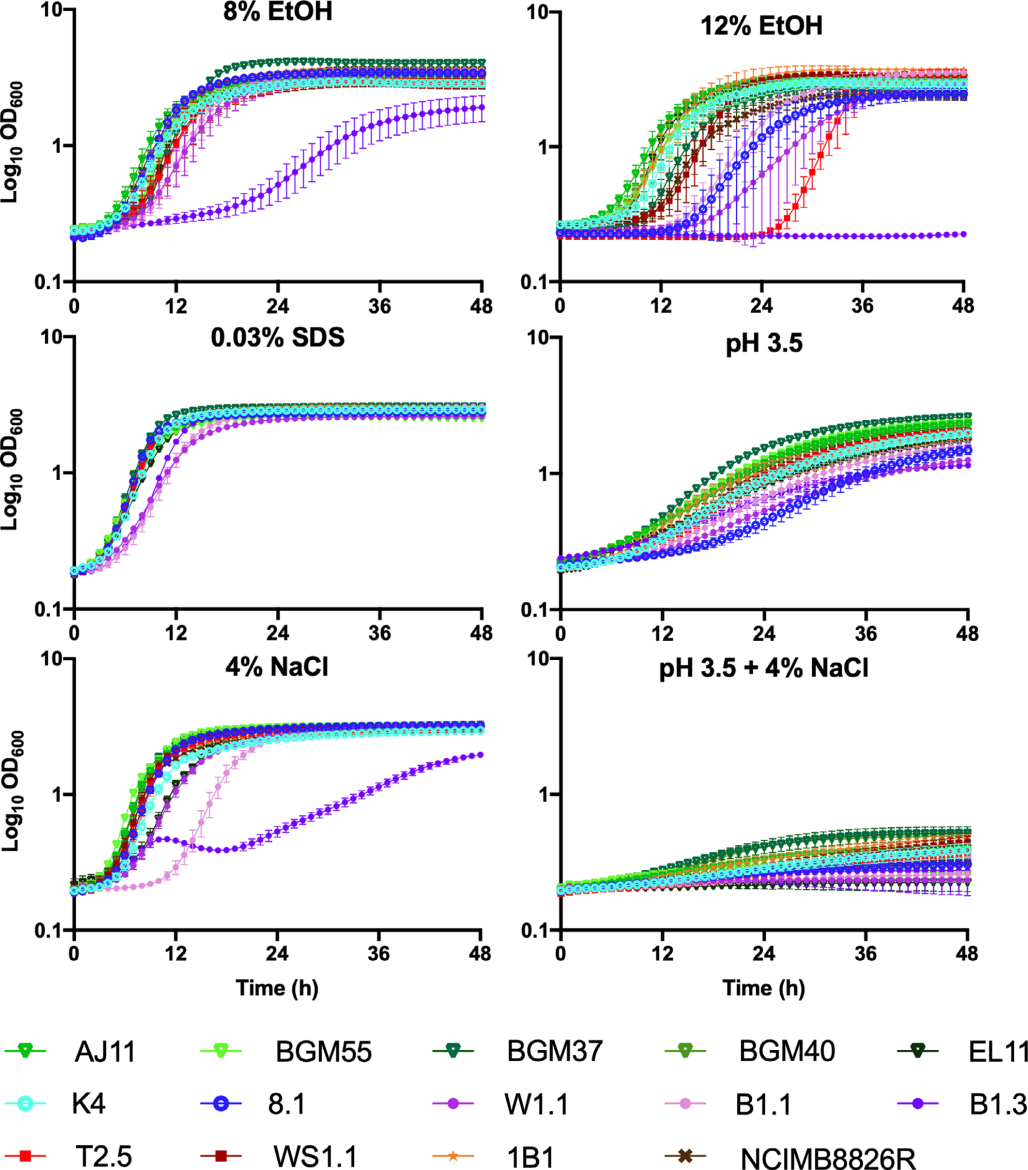
Growth of *Lactiplantibacillus* *plantarum* in mMRS‐glucose exposed to different environmental stresses. *L. plantarum* was incubated in mMRS‐glucose containing 8% (v/v) ethanol (EtOH), 12% (v/v) ethanol, 0.03% (w/v) SDS or 4% (w/v) NaCl with or without adjustment to pH 3.5 and incubated at 30°C for 48 h. The avg ± stdev OD_600_ values of three replicates for each strain are shown.

Because mMRS‐glucose resulted in robust growth of the majority of *L. plantarum* strains investigated here, that culture medium was used for investigation of stress tolerance properties. In mMRS‐glucose containing 8% (v/v) (174 mM) ethanol (EtOH), the AUCs for all strains except B1.3 (teff injera) were either moderate or robust (Figs 2 and 4; Table S2). Although lag phase times were longer (data not shown) and growth rates were reduced when ethanol was included in the culture medium (Table S5), the growth curves of six strains [AJ11, BGM37 and EL11 (fermented olives), 8.1 (wheat boza), B1.1 (teff injera) and 1B1 (cactus fruit)] were still regarded as robust according to AUC assessments (Fig. 2). Surprisingly, two strains, BGM37 (fermented olives) and 1B1 (cactus fruit), reached a higher final OD_600_ in mMRS‐glucose with 8% (v/v) ethanol than in mMRS‐glucose alone (Student’s *t*‐test, *P* < 0.05; Table S3).

None of the *L. plantarum* strains tested here were able to grow over a 48 h period when incubated directly in mMRS‐glucose with 12% (v/v) (260 mM) ethanol (data not shown). To determine whether a more gradual exposure to high ethanol concentrations would change this outcome, the strains were incubated in mMRS‐glucose containing 8% (v/v) ethanol overnight prior to inoculation into mMRS‐glucose with 12% (v/v) ethanol. This modification resulted in robust growth of 1B1 (cactus fruit; Figs 2 and 4; Tables S2, S3 and S5). Eight other strains [AJ11, BGM55, BGM37, BGM40 and EL11 (fermented olives), K4 (wheat sourdough), B1.1 (teff injera) and WS1.1 (fermented tomatoes)] exhibited moderate growth according to AUC values as a result of the stepwise transfer to the higher [12% (v/v)] ethanol conditions (Figs 2 and 4; Tables S2, S3 and S5).

### Growth in the presence of detergent (SDS) stress

While most of the *L. plantarum* strains exhibited moderate growth when sodium dodecyl sulfate (SDS) [0.03% (w/v) (0.10 mM)] was included in mMRS‐glucose, two strains BGM37 (fermented olives) and 1B1 (cactus fruit) grew robustly (Figs 2 and 4; Tables S2, S3 and S5). Remarkably, the growth rate of strain B1.3 (teff injera) was higher in the presence of SDS (0.32 ± 0.003 h^−1^) (Table S5) as opposed to its absence (0.20 ± 0.003 h^−1^) (Table S4) and it reached a higher AUC (107 ± 0.17) (Table S2).

### Growth at pH 3.5 and in the presence of 4% NaCl

Growth of *L. plantarum* was reduced in mMRS‐glucose adjusted to a pH of 3.5 (Figs 2 and 4; Table S2). However, the strains isolated from brine‐based, fruit fermentations [AJ11, BGM55, BGM37, BGM40 and EL11 (fermented olives) and T2.5 and WS1.1 (fermented tomatoes)] grew significantly better under those conditions compared to the *L. plantarum* isolated from grain fermentations (Student’s *t*‐test, *P* < 0.05). The strains from grain‐based fermentations [K4 (wheat sourdough), 8.1 (wheat boza), W1.1, B1.1 and B1.3 (teff injera)] grew poorly in the acidified mMRS (pH 3.5) (Figs 2 and 4; Table S2), yielding low growth rates (0.06 ± 0.01 h^−1^) (Table S5) and final OD_600_ values (1.50 ± 0.19) (Table S3).

When 4% (w/v) NaCl was included in mMRS‐glucose, five strains isolated from different sources [BGM55, BGM37 and BGM40 (fermented olives), 8.1 (wheat sourdough) and WS1.1 (fermented tomatoes)] were classified as robust according to their AUC values (Fig. 2; Table S2). The growth of strain B1.3 (teff injera) was the most negatively impacted by the addition of salt into the laboratory culture medium (Fig. 4; Tables S2, S3 and S5).

All *L. plantarum* strains were inhibited in mMRS‐glucose containing 4% (w/v) NaCl and a starting pH of pH 3.5 (Figs 2 and 4; Table S2). The final OD_600_ values ranged from a low of 0.23 ± 0.00 (W1.1, teff injera) to a high of 0.52 ± 0.06 (BGM37, fermented olives) (Table S3). Although the AUCs of all strains were regarded to be poor, growth rates of those isolated from brine‐based, fruit fermentations [AJ11, BGM55, BGM37, BGM40 and EL11 (fermented olives) and T2.5 and WS1.1 (fermented tomatoes)] were significantly higher than those isolated from grain‐based fermentations [K4 (wheat sourdough), 8.1 (wheat boza), W1.1, B1.1, and B1.3 (teff injera)] (*P* < 0.05, Student’s *t*‐test).

### Survival at pH 2

**Fig. 5:**
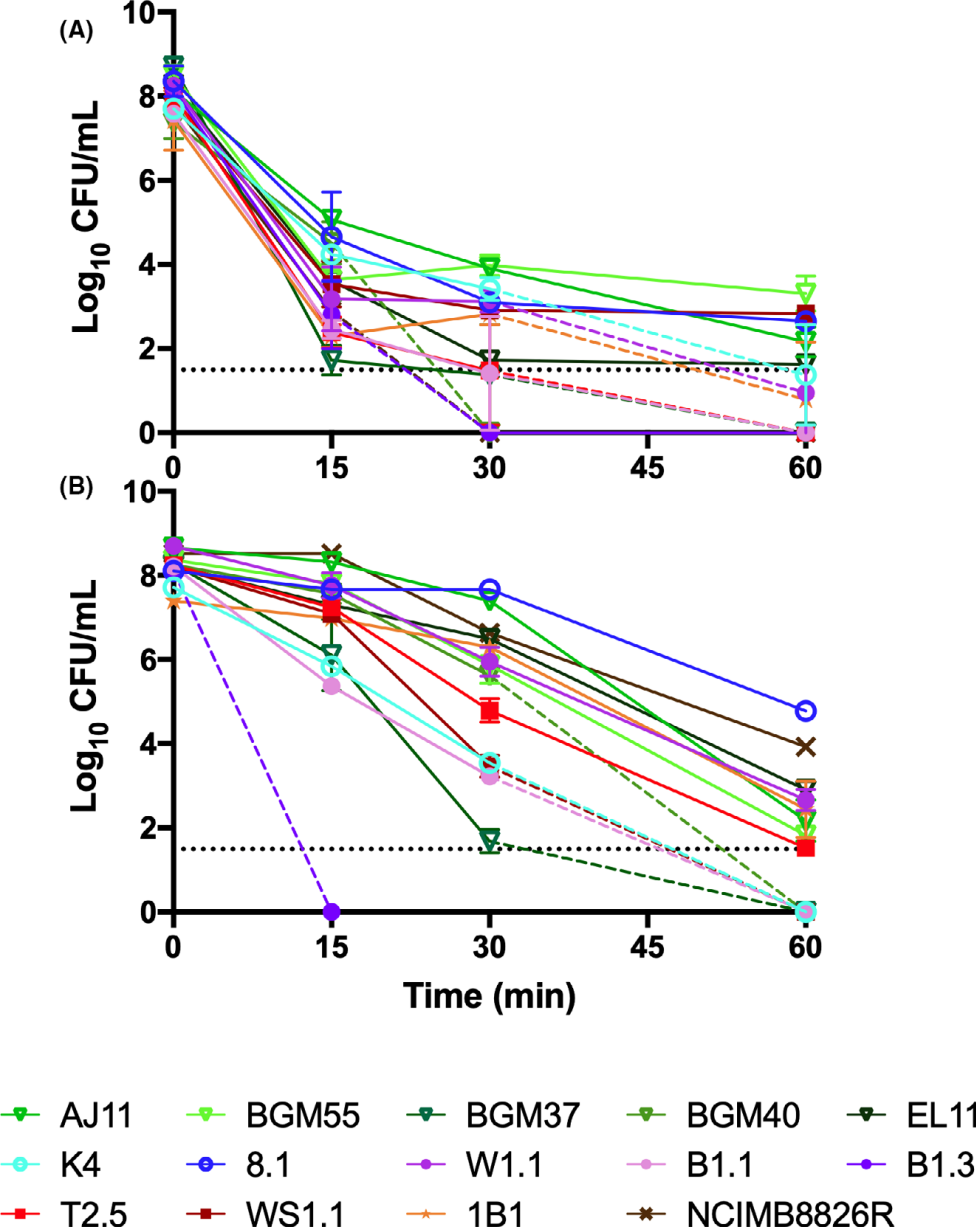
Survival of *Lactiplantibacillus* *plantarum* at (A) pH 2 and at (B) 50°C. (A) Viable cells were enumerated after 0, 15, 30 and 60 min of incubation in physiological saline at pH 2 or (B) in PBS at 50°C. The dashed lines indicate when the number of viable cells was below the detection limit (34 CFU ml^−1^). The avg ± stdev CFU ml^−1^ values of three replicates for each strain are shown.

Within 15 min incubation in physiological saline adjusted to pH 2, a 10^4^‐ to 10^6^‐fold reduction in cell viability was observed (Fig. 5A). After 30 min exposure to pH 2, strains B1.3 (teff injera), BGM40 (fermented olives) and NCIMB8826R (saliva, reference strain) were no longer detectable by colony enumeration. BGM37 (fermented olives), B1.1 (teff injera) and T2.5 (fermented tomatoes) were no longer viable by 60 min (Fig. 5A). *L*. *plantarum* AJ11, BGM55 and EL11 (fermented olives), 8.1 (wheat boza) and WS1.1 (fermented tomatoes) exhibited the highest acid tolerance and were still viable according to colony enumerations performed on cells collected after 60 min incubation. Unlike the findings for growth under acidic conditions (pH 3.5) (Fig. 4), there were no obvious isolation‐source dependent trends in *L. plantarum* strain survival.

### Survival at 50°C

Survival of *L. plantarum* at 50°C spanned a 10^6^‐ fold range (Fig. 5B). Viable B1.3 (teff injera) cells were no longer detected after incubation at 50°C for 15 min (1 × 10^8^ cells ml^−1^ present in the inoculum). After 60 min, AJ11 and EL11 (fermented olives), 8.1 (wheat boza), W1.1 (teff injera), 1B1 (cactus fruit) and NCIMB8826R (saliva, reference strain) were still culturable in a range from 5 × 10^4^ (8.1) to 1.5 × 10^2^ (AJ11) CFU ml^−1^, spanning a 10^3^‐ to 10^6^‐fold reduction in viable cell numbers (Fig. 5B). Similar to survival to pH 2, no obvious isolation‐source dependent differences in survival were observed.

### Biofilm forming capacity

**Fig. 6:**
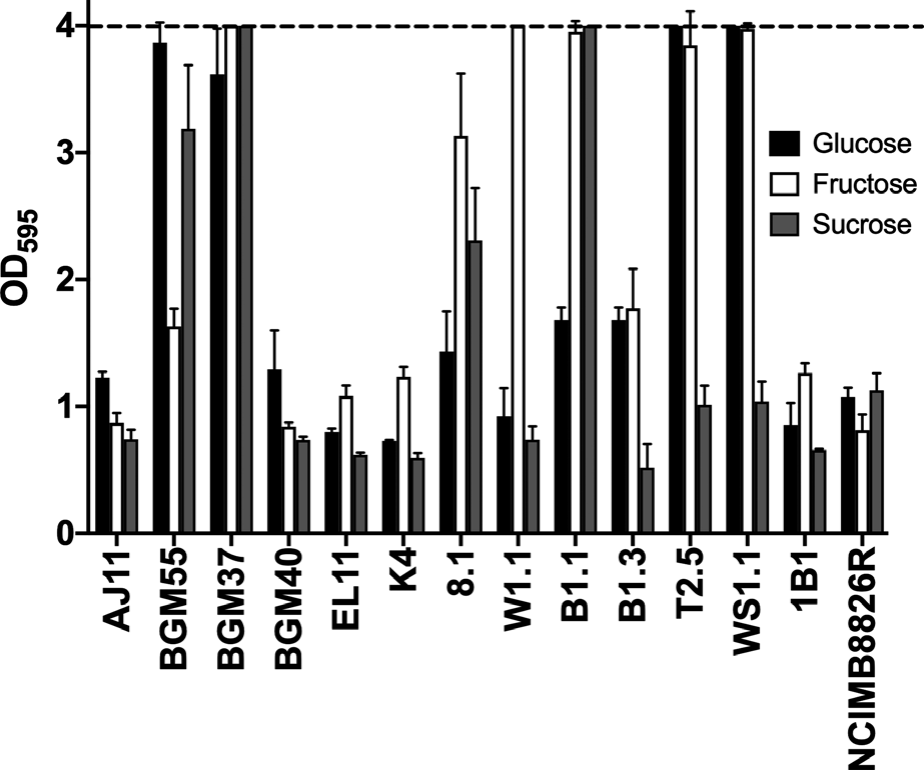
*Lactiplantibacillusplantarum* biofilm formation during growth in mMRS with glucose, fructose or sucrose. *L. plantarum* was incubated in mMRS‐glucose, mMRS‐fructose and mMRS‐sucrose in 96‐well, polystyrene microtiter plates at 30°C for 48 h. The non‐adherent cells were removed by washing with PBS. The remaining cells were stained with 0.05% crystal violet (CV). OD_595_ values of wells without cells did not exceed 0.22. The upper detection limit as indicated by the stippled line was an OD_595_ of 4.0. The avg ± stdev OD_595_ values of three replicate wells after CV staining are shown.

Because biofilm formation is an indicator of bacterial capacities to tolerate environmental stress (Yin *et al*., 2019) and *L. plantarum* biofilm formation is partially dependent on carbon source availability (Fernández Ramírez *et al*., 2015), we examined the capacity of *L. plantarum* to produce biofilms during growth in mMRS with glucose, fructose or sucrose. Only BGM55 and BGM37 (fermented olives), 8.1 (wheat boza), W1.1 and B1.1 (teff injera), T2.5 and WS1.1 (fermented tomatoes) formed robust biofilms after growth in at least one of those laboratory culture media (Fig. 6). Whereas injera strain W1.1 only developed a biofilm when grown in mMRS‐fructose, the other isolates formed robust biofilms in the presence of at least two different sugars (Fig. 6). Both strains isolated from fermented tomatoes, T2.5 and WS1.1, formed extensive biofilms when grown in the presence of either glucose or fructose. Notably, biofilm formation was not associated with robust strain growth. Strain 8.1 formed a biofilm in mMRS‐sucrose (Fig. 6) despite showing poor growth (Fig. 2) and reaching a low final OD_600_ (Table S3) in that culture medium. Conversely, strain K4 grew well in mMRS‐sucrose but did not produce a biofilm.

### Antifungal activity of *L. plantarum* cell‐free culture supernatant (CFCS)

**Fig. 7:**
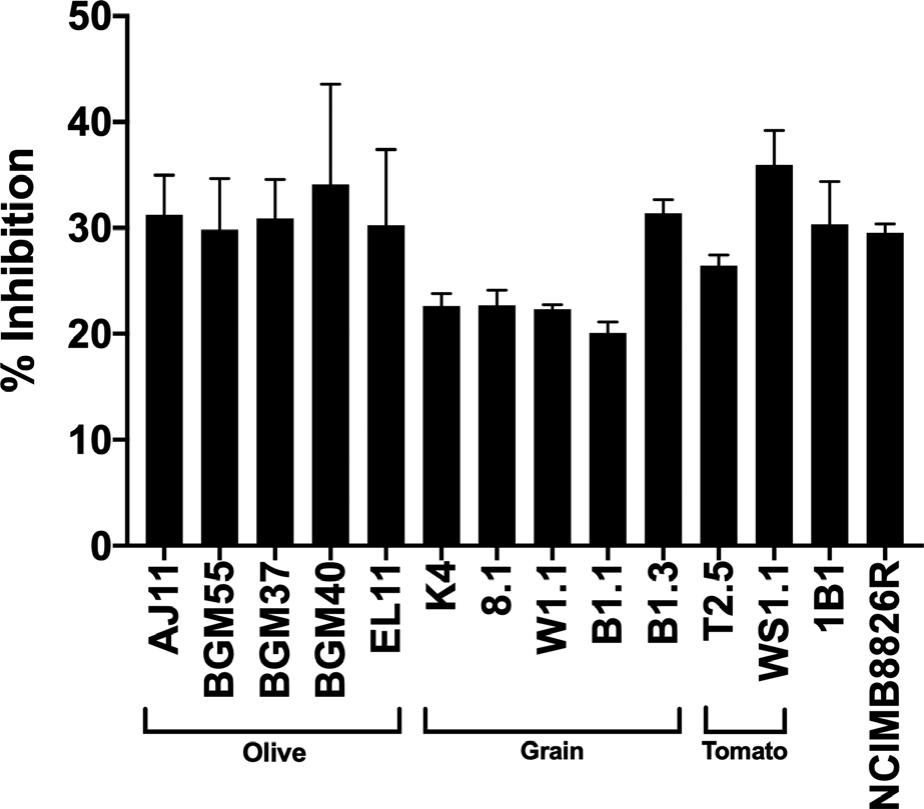
*Saccharomycescerevisiae* growth inhibition in the presence of *Lactiplantibacillus* *plantarum* CFCS. *S. cerevisiae* UCDFST‐09‐448 was incubated in a 1:1 ratio of 2X YM and pH adjusted *L. plantarum* CFCS from cMRS (pH 3.8). Growth was measured by monitoring the change in OD_600_ over 24 h. Per cent inhibition was determined by comparing the final OD_600_ of *S. cerevisiae* incubated in the presence of CFCS to cells incubated in a 1:1 ratio of 2X YM and pH adjusted (pH 3.8) cMRS (pH 3.8).

Growth rates and final OD_600_ values of *S. cerevisiae* UCDFST 09‐448 were reduced when incubated in the presence of the *L. plantarum* CFCS (Table S6). All *L. plantarum* CFCSs inhibited *S. cerevisiae* growth; however, there were some strain‐specific differences (Fig. 7; Table S6). Collectively, the CFCSs from strains isolated from fermented olives (AJ11, BGM55, BGM37, BGM40, EL11) and fermented tomatoes (WS1.1 and T2.5) were significantly (*P* < 0.05, Student’s *t*‐test) more inhibitory than those isolated from fermented grains (K4, 8.1, W1.1, B1.1 and B1.3). Among the strains isolated from fermented olives, growth inhibition resulting from exposure to the CFCS ranged between 29.8% ± 4.87 (BGM55) and 34.1% ± 9.4 (BGM40). By comparison, growth inhibition with CFCS from most *L. plantarum* isolated from grain fermentations was only between 20.1% ± 1.06 (B1.1) and 22.68% ± 1.46 (8.1). Interestingly, the growth pattern of *S. cerevisiae* in the presence of teff injera strain B1.3 CFCS (31.4 ± 1.27) was more similar to strains from fermented olives than grains.

### Comparisons of *L. plantarum* genomes

Nine of the fourteen strains were selected for genome sequencing (PacBio or Illumina platforms) based on the variations in their phenotypic profiles (Fig. 2). Genome assembly for strains sequenced using PacBio resulted in fewer contigs (min of 3 and max of 9) and higher coverage (min of 140X and max of 148X) compared to Illumina (contigs: min of 29 and max of 120; coverage (min of 27X and max of 128X) (Table 2). Genome sizes ranged from 3.09 Mbp [B1.3 (teff injera)] to 3.51 Mbp [WS1.1 (fermented tomatoes)], and total numbers of predicted coding sequences ranged from 3088 [K4 (wheat sourdough)] to 3613 (WS1.1) (Table 2).

**Table 2:** *Lactiplantibacillusplantarum* genome coverage and assembly statistics.

Strain^a^	Accession No.	Genome size (Mb)	# of Contigs	Coverage	N50	L50	% GC content	# of CDS
AJ11	WWDD00000000	3.27	29	27X	252487	6	44.54	3214
BGM37	WWDC00000000	3.46	46	66X	155998	7	44.15	3467
EL11	WWDB00000000	3.28	29	128X	1944449	5	44.31	3231
K4	WWDF00000000	3.16	3	148X	3157988	1	44.60	3088
8.1	WWDE00000000	3.37	9	140X	3066287	1	44.40	3366
B1.1	WWCZ00000000	3.17	120	76X	59472	19	44.55	3242
B1.3	WWCY00000000	3.09	5	145X	2939357	1	44.50	3157
WS1.1	WWDA00000000	3.51	99	30X	78600	12	44.11	3613
1B1	WWDG00000000	3.34	60	28X	109565	11	44.34	3371

^a^ The genomes of AJ11, EL11, BGM37, WS1.1 and 1B1 were sequenced by Illumina MiSeq V2 (2 × 250). The genomes of strains K4, 8.1 and B1.3 were sequenced by PacBio RSII (P6‐C4 sequencing chemistry).

The core‐ and pan‐genomes of the nine strains consisted of 2222 and 6277 genes, respectively (Fig. S5), numbers consistent with previous comparisons examining larger collections of *L. plantarum* strains (Siezen *et al*., 2010; Martino *et al*., 2016; Choi *et al*., 2018). Alignments of the predicted amino acid sequences for the genes in the core genomes indicated that strains isolated from grain fermentations [K4 (wheat sourdough), 8.1 (wheat boza) and B1.1, and B1.3 (teff injera)] and strain WS1.1 from fermented tomatoes are more closely related to each other than isolates from olives and cactus fruit (Fig. 1B). B1.1 and B1.3, two strains originating from the same sample of teff injera, were also shown to share similar core genomes (Fig. 1B).

**Fig. 8:**
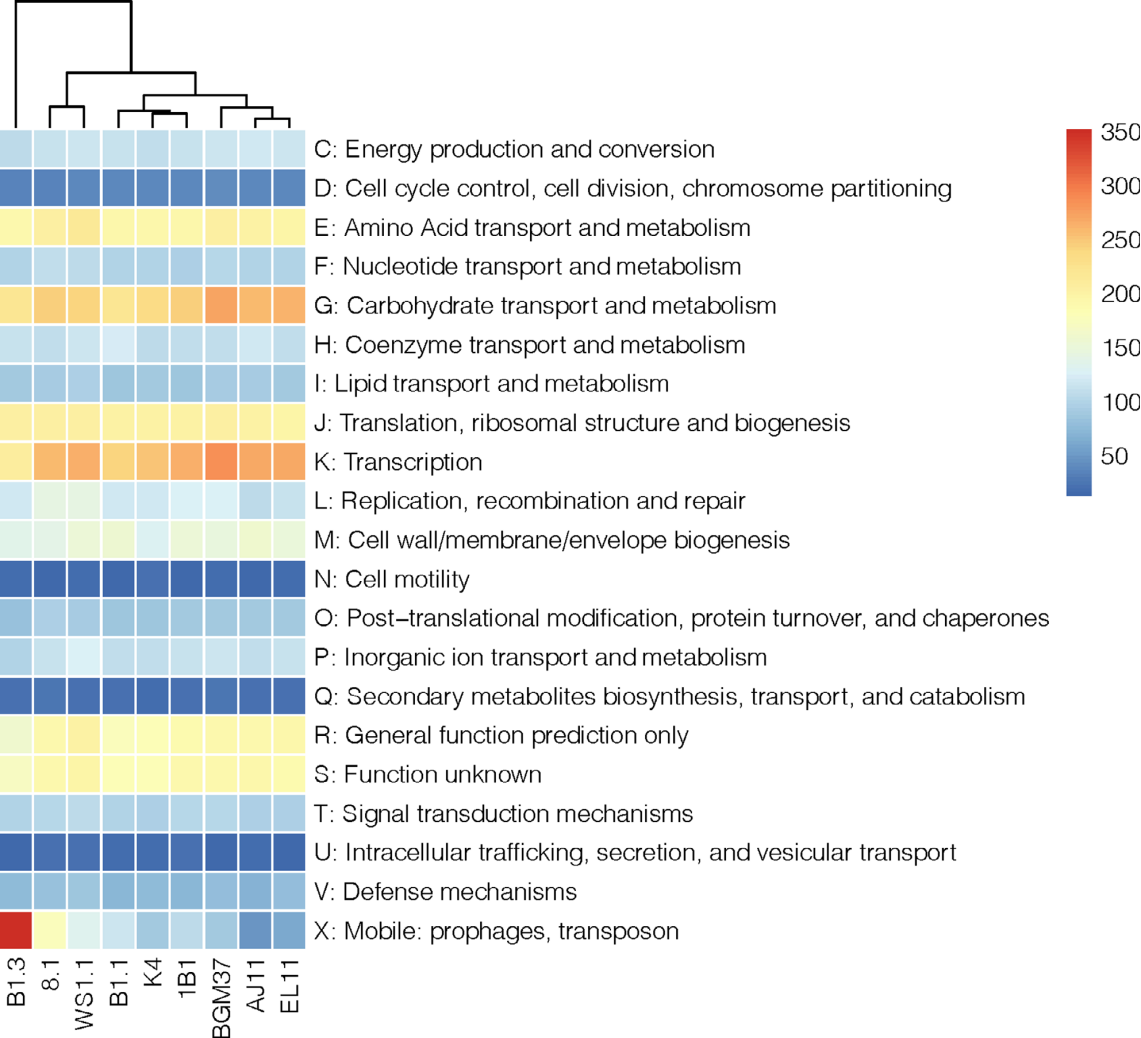
Distribution of COG Categories across *Lactiplantibacillus* *plantarum* genomes. Hierarchical clustering of *L. plantarum* based on the number of gene clusters assigned to each functional COG category. Number of gene clusters present in each strain is indicated by the colour gradient.

Just as strains 8.1 (wheat boza) and WS1.1 (fermented tomatoes) were found to have similar core genomes (Fig. 1B), those two strains are similar according to hierarchical clustering based on the numbers of genes in individual cluster of orthologous group (COG) categories (Fig. 8). The three strains isolated from olives formed a separate clade from those recovered from other sources and were shown to have higher numbers of genes in the carbohydrate metabolism and transport (G) and transcription (K) COGs. *L. plantarum* BGM37, a strain from olives that exhibited the most robust growth on the different carbohydrates compared tested here (Fig. 2), also contained the highest numbers of gene clusters annotated to the carbohydrate metabolism and transport COG (256 gene clusters, Fig. 8; Table S7).

Strain B1.3 was found to contain the lowest number of gene clusters in the carbohydrate metabolism and transport COG (206 gene clusters, Fig. 8; Table S7) and is specifically lacking numerous genes required for sugar metabolism and sugar‐importing phosphotransferase (PTS) systems (data not shown). Conversely, the genome of B1.3 harbours at least twofold higher numbers of genes and genetic elements in the mobilome (X) COG compared to the other strains examined (352 gene clusters, Table S7). These genomic features include prophages, insertion sequence elements and transposases that are interspersed throughout the genome and frequently located between genes with known function. For example, a transposon (3.8 kb) is located between the glucose‐6‐phosphate isomerase (lp_2502) and glucose/ribose porter family sugar transporter (lp_2503) genes that are annotated to be associated with glucose metabolism. Other genes were not present in the B1.3 genome such as the sucrose‐associated PTS (lp_3819; *pts24BCA*), possibly indicating why this strain exhibited poor growth in mMRS‐sucrose. Strain 8.1, the only other *L. plantarum* strain tested here that grew to a limited extent on sucrose (Fig. 2), lacks the first 650 bp of *pts1BCA* (lp_0185), a gene in the sucrose phosphoenolpyruvate (PEP)‐dependent phosphotransferase system (PTS; Saulnier *et al*., 2007; Yin *et al*., 2018).

The number of gene clusters in the other COG categories was largely conserved between strains (Fig. 8; Table S7). These COG categories encode pathways required energy metabolism (glycolysis), synthesis of macromolecules (proteins, nucleotides and lipids) and stress response. The genomes of all nine strains contain genes encoding chaperones (DnaJK, GroEL, GroES, GrpE, ClpB, ClpL), proteases (ClpX, ClpP, ClpE), DNA repair proteins (RecA, UvrABC) and transcriptional regulators (HrcA, CtsR) critical for *L. plantarum* tolerance to numerous environmental stresses (Papadimitriou *et al*., 2016). Although genes required for citrate metabolism (*citCDEF*) were previously found to be associated with ethanol tolerance (van Bokhorst‐van de Veen *et al*., 2011) and that locus was flanked by mobile elements in several of the *L. plantarum* strains examined here, the presence of those mobile elements was not correlated with ethanol sensitivity.

## Discussion

This study investigated the phenotypic and genetic properties of *L. plantarum* strains from (fermented) plant sources. The findings broadly show that strains obtained from the same or similar plant environments tend to be more genetically related and share similar carbohydrate utilization and stress tolerance capacities. However, there were still significant differences between all strains, irrespective of their source, a result which suggests that *L. plantarum* has adapted for growth in specific habitats (e.g. olive fermentations) but that intraspecific variation of this generalist species may afford the opportunity for *L. plantarum* strain coexistence by niche differentiation.

Our use of growth curve area under the curve (AUC) rankings and the monitoring of growth rates and final OD_600_ values provided a detailed view of *L. plantarum* carbohydrate utilization capacities. The majority of strains exhibited robust growth on glucose, maltose, sucrose and galactose, moderate growth on raffinose and fructose, and only limited to no growth on ribose, arabinose and xylose. The moderate or poor growth observed for a few strains when incubated the presence galactose or fructose was likely not due to carbon catabolite repression (Görke and Stülke, 2008; Kremling *et al*., 2015), but rather a lack of enzymatic capacity to utilize those sugars. These conserved carbohydrate consumption patterns are consistent with prior reports on *L. plantarum* isolated from plants and other host‐associated sources (Westby *et al*., 1993; Saulnier *et al*., 2007; Siezen *et al*., 2010; Filannino *et al*., 2014; Siragusa *et al*., 2014). The strains tested here were also able to grow in the presence of 0.03% (w/v) SDS and were severely impaired when incubated in mMRS at pH 3.5 with 4% (w/v) NaCl or inoculated directly into mMRS with 12% (v/v) ethanol.

Other findings were strain‐specific and similarly consistent with reported phenotypic (Parente *et al*., 2010; Siezen *et al*., 2010; Guidone *et al*., 2014; Ferrando *et al*., 2015, 2016; Gheziel *et al*., 2019; Fuhren *et al*., 2020; Prete *et al*., 2020) and genomic variations (Molenaar *et al*., 2005; Siezen *et al*., 2010; Siezen and van Hylckama Vlieg, 2011; Martino *et al*., 2016; Choi *et al*., 2018; Cen *et al*., 2020; Pérez‐Díaz *et al*., 2021) observed for the *L. plantarum* species. We found that *L. plantarum* growth was highly variable following the sequential incubation in 8% (v/v) and then 12% (v/v) ethanol. Strain growth rates in mMRS with 8% (v/v) ethanol were correlated with those observed for mMRS containing 0.03% SDS (*r* = 0.561, *P* < 0.05), thereby indicating overlapping mechanisms in *L. plantarum* strain tolerance to membrane‐disruptive compounds (Seddon *et al*., 2004; Bravo‐Ferrada *et al*., 2015; Mukhopadhyay, 2015). High temperature tolerance also differed between the *L. plantarum* isolates, such that incubation at 50°C for 60 min resulted in over a 10^5^‐fold range in strain survival. Survival at pH 2 followed a similar trend, such that some strains were no longer culturable after 15 min, while other strains still formed colonies after prolonged (60 min) incubation. Notably, only two strains from olive fermentations (AJ11 and EL11) and 8.1 from boza survived well under both high temperature and low pH conditions. Although, the genomes were found contain chaperones and proteases known to be involved in *L. plantarum* heat and acid shock responses (Corcoran *et al*., 2008; Mills *et al*., 2011), the unique proteins or pathways expressed by those strains which confer heightened stress tolerance remain to be determined.

Despite the conserved and variable aspects of *L. plantarum* carbohydrate utilization and environment stress tolerance phenotypes, there were other remarkable trends associated with strain isolation source. For example, the isolates from acidic, brine‐containing ferments (olives and tomatoes) were more resistant to acidic pH (pH 3.5) and high NaCl (4% w/v) concentrations than those recovered from grain fermentations (wheat boza, wheat sourdough and teff injera). Genome comparisons using concatenated core gene amino acids showed that strains isolated from grain fermentations are more related to each other than those from other sources. Genetic conservation between olive fermentation‐associated strains was observed by MLST and COG gene numbers. These results are in agreement with a recent comparative genomics study of 140 strains of *L. plantarum* isolated from variable environments, which found that strains isolated from similar ecological niche share similar functional profiles (Cen *et al*., 2020).

The strains from fermented olives also showed the greatest capacity to consume raffinose (a tri‐saccharide composed of galactose, glucose and fructose). It is also notable that two of those isolates (BGM37 and BGM55) grew equally well in mMRS‐galactose as in mMRS‐glucose. These results are consistent with the findings that olives leaves and roots contain both raffinose (2.7 ± 0.1 µmol) and galactose (4.8 ± 0.3 µmol) (Cataldi *et al*., 2000) and that the fruits contain galactose along with higher concentrations of glucose, mannitol and fructose (Gómez‐González *et al*., 2010). All strains from olive fermentations also exhibited at least moderate or robust growth in mMRS in the presence of 8% (v/v) ethanol, and the CFCSs from those strains resulted in greater inhibition of *S. cerevisiae* UCDFST‐09‐448 compared to the CFCSs from *L. plantarum* isolated from other environments. Because yeast are normal members of olive fermentation microbiota, the inhibitory capacity may indicate the presence of shared mechanisms required to prevent yeast overgrowth.

Several strains also showed unique properties illustrative of the phenotypic range of the *L. plantarum* species. Among those strains was BGM37 isolated from the brine of fermented olives. This strain exhibited the most robust growth on the carbohydrates tested here, showed the highest tolerance to 8% ethanol and 0.03% SDS and was able to form biofilms in the presence of glucose, fructose and sucrose. Compared to the other strains for which genome sequences were obtained, BGM37 was found to have the second largest genome size (3.46 Mbp) after WS1.1 (3.51 Mbp), a magnitude comparable to the other *L. plantarum* strains with large (complete) genomes published at NCBI (maximum of 3.70 Mbp as of Jan 2021).

*Lactiplantibacillusplantarum* 1B1, a strain isolated from ripe cactus fruit, is notable because of its robust growth in the presence of either SDS or ethanol. Although other studies reported growth of *L. plantarum* in the presence of ethanol (van Bokhorst‐van de Veen *et al*., 2011; Brizuela *et al*., 2019; Chen *et al*., 2020), the capacity to grow well at 12% ethanol is an unusual trait even among oenological‐associated *L. plantarum* (Succi *et al*., 2017). Thus, the unique properties of this single isolate from a fresh fruit source may indicate the presence of a broader diversity of LAB present in the carposphere (Yu *et al*., 2020).

Lastly, strain B1.3 from teff injera exhibited the most restrictive carbon utilization capacities and the lowest levels of environmental stress tolerance among all isolates tested. B1.3 grew poorly on glucose and most other carbohydrates, whereas the other strains from teff injera B1.1 and W1.1 exhibited robust growth on a variety of sugars. Limitations in the ability of B1.3 to consume different sugars were also shown by the lower numbers of gene clusters in the B1.3 genome that are responsible for carbohydrate transport and metabolism. The overall smaller genome size of this strain (3.09 Mbp) and high numbers of genes in the mobilome COG potentially indicates that this strain is undergoing genome reduction for habitat specialization as found for other LAB [e.g. *Lactobacillus bulgaricus* (yoghurt) (van de Guchte *et al*., 2006), *Lactobacillus iners* (vagina) (France *et al*., 2016) and *Apilactobacillus apinorum* (honeybee) (Endo *et al*., 2018)]. Remarkably, the higher growth rate of B1.3 in mMRS‐fructose and in the presence of SDS indicates it may be fructophilic and capable of withstanding the presence of membrane disrupting compounds in teff flour. The finding that the CFCS from B1.3 inhibited *S. cerevisiae* UCDFST 09‐448 growth also suggests that B1.3 may be adapted to compete with yeast in teff injera. This result is consistent with the proximity of B1.3 to the olive‐associated strains in the MLST phylogenetic tree. However, it is also noteworthy that B1.3 shares genetic similarity with the teff injera isolate (B1.1) and other grain‐associated *L. plantarum* according to core genome comparisons.

Although disruptions in sucrose PTS systems may indicate why neither strain B1.3 nor 8.1 was able to grow in the presence of sucrose, the specific genes and pathways conferring the phenotypic variations observed in this study remain to be determined. To this regard, identification of the genome composition alone is insufficient to understand the full metabolic and functional potential of this species. For example, there still remains a lack of resolution in some PTS and other carbohydrate transport and metabolic pathways among lactobacilli (Gänzle and Follador, 2012; Zheng *et al*., 2015) and stress response mechanisms frequently involve numerous pathways with overlapping cell functions (e.g. membrane synthesis, protein turnover and energy metabolism pathways; Papadimitriou *et al*., 2016).

The genetic and phenotypic variation observed for the *L. plantarum* isolates indicate this species has evolved towards specialization in different plant‐associated habitats (e.g. fruit vs cereal grains), but at the same time is under selective pressure for sustaining intraspecific diversity within those habitats, possibly as a mechanism promoting *L. plantarum* species stability through co‐occurrence in those ecosystems (Maynard *et al*., 2019). This can be investigated using *L. plantarum* strains possessing shared and variable traits in plant and fermented plant food colonization assays. Overall, these efforts will be useful for understanding bacterial interactions and habitat partitioning in other complex host‐associated (e.g. Lloyd‐Price *et al*., 2017; Truong *et al*., 2017; Bongrand and Ruby, 2019; Ma *et al*., 2020) and environmental (e.g. Ellegaard *et al*., 2015; Koch *et al*., 2020; Props and Denef, 2020) sites wherein significant intraspecies diversity has been found but not yet understood. These findings may also be used to guide the selection of robust, multi‐strain starter cultures that are suited to inter‐ and intraspecies selection pressures in fruit and vegetable fermentations to result in optimal sensory and safety characteristics.

## Experimental procedures

### Bacterial strains and growth conditions

*Lactiplantibacillusplantarum* strains used in this study are shown in Table 1. The isolates from olive fermentations and cactus fruit were described previously (Golomb *et al*., 2013; Tyler *et al*., 2016), and NCIMB8826R, a rifampicin‐resistant variant (Yin *et al*., 2018) of strain NCIMB8826 (Hayward and Davis, 1956), was used as a reference. For *L. plantarum* isolation from injera batter, the batter was mixed with phosphate buffered saline (PBS, 137 mM NaCl, 2.7 mM KCl, 4.3 mM Na_2_HPO_4_‐7H_2_O, 1.4 mM KH_2_PO_4_) (pH 7.2) at a ratio of 1:10. For isolation from boza and sourdough, the batter was mixed with physiological saline (145 mM NaCl) (pH 7.0) at a ratio of 1:10. For isolation from fermented tomatoes, three tomatoes were placed in sterile bags containing mesh filters (Nasco, Modesto, CA) with 1 ml of PBS (pH 7.2) and macerated by hand. Serial dilutions of the injera, boza, sourdough and tomato suspensions were then plated on de Man, Rogosa and Sharpe (MRS) agar from a commercial source (BD, Franklin Lakes, NJ) (cMRS). Natamycin (25 μg ml^−1^) (Dairy Connection, Wisconsin, WI) was included in the cMRS agar to inhibit fungal growth. The cMRS agar plates were incubated at 30°C under aerobic or anaerobic conditions [BD BBL GasPak system (BD, Franklin Lakes, NJ)] for 48 h. Single colony isolates were repeatedly streaked for isolation on cMRS prior to characterization. For phenotypic and genotypic analysis, the *L. plantarum* strains were routinely grown in cMRS without aeration at 30°C.

### Strain identification and typing

*Lactiplantibacillusplantarum* 16S rRNA genes were amplified from individual colonies using the 27F and 1492R primers (Lane *et al*., 1991; Table S8) with *ExTaq* DNA polymerase (TaKaRa, Shiga, Japan). Thermal cycling conditions were as follows: 95°C for 3 min, 30 cycles of 94°C for 30 s, 50°C for 30 s and 72°C for 90 s, and a final elongation step of 72°C for 5 min. The PCR products were purified [Wizard SV Gel and PCR Clean‐Up System (Promega, Madison, WI)] and sequenced at the UC Davis DNA Sequencing Facility http://dnaseq.ucdavis.edu/. The DNA sequences were compared against the National Center for Biotechnology Information (NCBI) database using the nucleotide Basic Local Alignment Search Tool (Blastn; https://blast.ncbi.nlm.nih.gov/Blast.cgi) and the Ribosomal Database Project (RDP; http://rdp.cme.msu.edu/). Multiplex PCR targeting the *recA* gene was also used to confirm *L. plantarum* at the species level according to methods described by (Torriani *et al*., 2001; Table S8). The 16S rRNA sequencing data for the strains in this study can be found National Center for Biotechnology Information (BankIt) under accession numbers MT937284‐MT937296.

For multi‐locus sequence typing (MLST), genomic DNA was isolated with the Qiagen DNeasy Blood and Tissue Kit (Qiagen, Valencia, CA) according to the manufacturer’s instructions. PCR was then performed using primers targeting the variable regions of *L. plantarum pheS*, *pyrG*, *uvrC*, *recA*, *clpX*, *murC*, *groEL* and *murE* (Table S8; Xu *et al*., 2014). PCR amplification was preformed using *ExTaq* DNA polymerase (TaKaRa, Shiga, Japan) as previously described (Xu *et al*., 2014). The PCR products were sequenced in both directions using the forward and reverse primers at the UC Davis DNA Sequencing Facility (http://dnaseq.ucdavis.edu/) and Genewiz (South Plainfield, NJ). DNA sequences were aligned, trimmed and analysed using the Mega 7.0 software package (Kumar *et al*., 2016). Based on the findings, unique nucleotide sequences for a gene were defined as an allele and unique allelic profiles were defined as a sequence type. The concatenate sequences in the order of *pheS*, *pyrG*, *uvrC*, *recA*, *clpX*, *murC*, *groEL* and *murE* was used for phylogenetic tree analysis with maximum likelihood supported with a multi‐locus bootstrap approach using Mega 7.0 (Kumar *et al*., 2016). For comparisons to other strains of *L. plantarum*, the sequences of 264 strains of *L. plantarum* were downloaded from the National Center for Biotechnology Information (NCBI) database (https://www.ncbi.nlm.nih.gov/), and a minimum spanning tree of the 278 strains was made using Phyloviz Online (Ribeiro‐Gonçalves *et al*., 2016). The MLST DNA sequences can be found in the National Center for Biotechnology Information (BankIt) under gene accession numbers MT864201–MT864291 and MT880889–MT880901.

### Genome sequencing, assembly, annotation and analysis

Nine strains were selected for genome sequencing by either Illumina MiSeq (Illumina, San Diego, CA) (B1.1, WS1.1, 1B1, AJ11, BGM37, EL11) or Pacific Biosciences (PacBio, Menlo Park, CA) (B1.3, 8.1, K4) DNA sequencing methods. For the Illumina MiSeq, approximately 3 − 10^9^ cells were suspended in lysis buffer containing 200 mM NaCl, 20 mM (ethylenediaminetetraacetic acid) EDTA, 500 µl of 793 mM sodium dodecyl sulfate (SDS) and 300 mg of zirconium beads (0.1 mm, BioSpec Products, Bartlesville, OK). The cells were then mechanically lysed by bead‐beating at 6.5 m/s for 1 min with a FastPrep‐24 (MP Biomedical, Santa Ana, CA). To obtain larger DNA fragments appropriate for PacBio DNA sequencing, total genomic DNA was extracted from each strain by incubating approximately 3 × 10^9^ cells in the presence of 20 mg ml^−1^ lysozyme (Sigma‐Aldrich, St. Louis, MO) at 37°C for 60 min. After extraction by either mechanical or enzymatic lysis, DNA was purified using phenol‐chloroform and ethanol precipitation methods (Sambrook and Russell, 2006).

Illumina libraries were prepared for paired‐end 250‐bp sequencing (2 × 250 bp) using the Nextera DNA Flex Library kit (Illumina, San Diego, CA). The libraries were sequenced at the UC Davis Genome Center (Davis, CA) (https://genomecenter.ucdavis.edu/) on an Illumina Miseq V2 according to the manufacturer’s protocol. Genomes were assembled with Spades (v3.12.0, using k‐mers 31, 51, 71), and Quast (v 4.6.3) was used to confirm assembly quality. The assembled genome sequences were then annotated with Rasttk (https://rast.nmpdr.org/) and PATRIC (Wattam *et al*., 2017). PATRIC comprehensive genome analysis was run using default auto parameters. This program encompasses BayesHammer for read error correction, Velvet, IDBA, and Spades for assembly, and ARAST to verify assembly quality (Wattam *et al*., 2017).

PacBio libraries were prepared and sequenced at the UC Davis Genome Center (Davis, CA) (https://genomecenter.ucdavis.edu/) on a Pacific Biosciences RSII instrument using P6‐C4 sequencing chemistry. Sequence SMRTcell files were imported into the PacBio SMRT portal graphical interface unit (https://www.pacb.com/) for de novo assembly using the hierarchical genome‐assembly process (HGAP) protocol (Chin *et al*., 2013) and RS HGAP Assembly 2 in Smart analysis version 2.3 software. The resulting assemblies were used for subsequent annotation with Rasttk (https://rast.nmpdr.org/) and Patric (Wattam *et al*., 2017). The whole genome sequencing data for this study can be found in the National Center for Biotechnology Information under the BioProject PRJNA598971.

Edgar 2.0 was used to evaluate the size of the pangenome and identify the number of genes shared between all nine sequenced strains as well as to identify the phylogenetic relationships between the different strains (Blom *et al*., 2016). The pan and core genomes were identified, and the results were presented as ortholog sets. To evaluate phylogenetic relationships, concatenate core amino acid sequences were aligned using Muscle (Edgar, 2004). The resulting alignment was used to construct a phylogenetic tree using a maximum likelihood method with bootstrapping in Mega 7.0 (Kumar *et al*., 2016). Anvi’o (v6.1) was used to group orthologous protein sequences into gene clusters for cluster of orthologues group (COG) functional assignments using the program ‘anvi‐pan‐genome’ (Eren *et al*., 2015; Delmont and Eren, 2018) with the flags ‘‐use‐ncbi‐blast’ (Altschul *et al*., 1990) and parameters ‘‐minibit 0.5’ (Benedict *et al*., 2014) and ‘mcl‐inflation 10’. COG frequency heat map with hierarchical clustering was generated using RStudio with the package ‘pheatmap’ (https://www.rstudio.com/). To confirm the truncation of *pts1BCA* in *L. plantarum* 8.1, the *pts1BCA* gene was amplified from genomic DNA from strains B1.3, K4, 8.1, and NCIMB8826R using the *pts1BCA_*trunF (5′‐ TCGTCACCGAGTGTTCGTTT) and *pts1BCA_*trunR (5′‐ AGTTGCTGGCCACTGTTCAT) primers (Table S8) and *ExTaq* DNA polymerase (TaKaRa, Shiga, Japan). Thermal cycling conditions were as follows: 95°C for 3 min, 30 cycles of 94°C for 30 s, 50°C for 30 s and 72°C for 90 s, and a final elongation step of 72°C for 5 min. PCR products were visualized on a 1% agarose gel.

### Carbohydrate utilization

*Lactiplantibacillusplantarum* strains were first incubated in cMRS for 24 h at 30°C. The cells were then collected by centrifugation at 5000× *g* for 5 min, washed twice in PBS to remove residual nutrients (pH 7.2) and then suspended in a modified MRS (mMRS) without beef extract or dextrose (pH 6.5) (De Man *et al*., 1960). The cell suspensions were then distributed into 96‐well microtiter plates (Thermo Fisher Scientific, Waltham, MA) at an optical density (OD) at 600 nm (OD_600_) of 0.2. To test the capacity to grow on different sugars, mMRS was amended to contain 2% (w/v) of d‐glucose (111 mM) (Fisher Scientific, Fair Lawn, NJ), d‐maltose monohydrate (55 mM) (Amresco, Solon, OH), sucrose (58 mM) (Sigma, St. Louis, MO), d‐galactose (111 mM) (Fisher Scientific, Fair Lawn, NJ), d‐raffinose pentahydrate (40 mM) (VWR International, Solon, OH), D‐fructose (55 mM) (Fisher Scientific, Fair Lawn, NJ), d‐xylose (133 mM) (Acros Organics, Morris Plains, NJ), d‐ribose (133 mM) (Acros Organics, Morris Plains, NJ) or l‐arabinose (133 mM) (Acros Organics, Morris Plains, NJ). The OD_600_ values were measured hourly for 48 h in a Synergy 2 microplate reader (Biotek, Winooski, VT) set at 30°C without aeration.

### Growth during exposure to ethanol, SDS, NaCl and pH 3.5

*Lactiplantibacillusplantarum* was incubated in cMRS for 24 h at 30°C. The cells were then collected by centrifugation at 5000× *g* for 5 min, washed twice in PBS (pH 7.2) and then suspended in mMRS‐glucose (2% (w/v) (111 mM) d‐glucose) (pH 6.5). The cell suspensions were then distributed into 96‐well microtiter plates containing mMRS‐glucose amended to contain ethanol [8% (v/v) (174 mM) or 12% (v/v) (260 mM)], SDS [0.03% (w/v) (0.10 mM)] or NaCl [4% (w/v) (68 mM)]. For measuring the effects of low pH, mMRS‐glucose was adjusted to pH 3.5 with 1 M HCl. For measuring the effect of both low pH and high NaCl concentration, mMRS‐glucose (pH 3.5) was supplemented with 4% (w/v) (68 mM) NaCl. Each strain was also incubated in mMRS diluted with water between [4 and 12% (v/v)] to control for dilution of mMRS due to amendment addition. The OD_600_ was used to monitor growth during incubation at 30°C for 48 h without aeration using a Synergy 2 microplate reader (Biotek, Winooski, VT).

### Survival at pH 2 or 50°C

For assessing acid tolerance, *L. plantarum* was incubated in cMRS for 24 h at 30°C prior to collection by centrifugation at 5000× *g* for 5 min and washing twice in physiological saline (145 mM NaCl) (pH 7.0). *L. plantarum* was then inoculated at a concentration of 1 × 10^8^ cells ml^−1^ in physiological saline adjusted to pH 2 with 5 M HCl in 1.5 ml tubes. Survival was measured after 0, 15, 30 and 60 min incubation at 30°C. At each time point, three tubes were retrieved per stain for centrifugation at 10 000× *g* for 1 min. The supernatant was discarded, and the resulting cell pellet was suspended in 1mL physiological saline (pH 7.0). Serial dilutions were then plated on cMRS agar and incubated at 30°C for 48 h prior to colony enumeration.

### Survival at 50°C

To measure thermal tolerance, *L. plantarum* was incubated in cMRS for 24 h at 30°C prior to collection by centrifugation at 5000× *g* for 5 min and washing twice in PBS (pH 7.2). The suspensions were then distributed into 0.2 ml tubes at approximately 1 × 10^8^ CFU ml^−1^ and incubated in a C1000 Thermal Cycler (Bio‐Rad Laboratories, Foster City, CA) at 50°C for 0, 15, 30 and 60 min. At each time point, three tubes were retrieved per strain. Serial dilutions of the cell suspensions were plated onto cMRS agar and incubated at 30°C for 48 h prior to colony enumeration.

### Biofilm formation assay

The potential for *L. plantarum* to form biofilms was assessed by measuring adherence to polystyrene according to previously described methods (Kopit *et al*., 2014) with several modifications. Briefly, 96‐well polystyrene plates (Thermo Fisher Scientific, Waltham, MA) containing either mMRS‐glucose, mMRS‐fructose or mMRS‐sucrose were inoculated with *L. plantarum* to a starting OD_600_ of 0.2 and the plates were incubated at 30°C for 48 h. The wells were then rinsed with PBS (pH 7.2), stained with 0.05% (w/v) crystal violet (CV), dried in an inverted position for 30 min and then rinsed again three times with PBS (pH 7.2). Absorbance at OD_595_ was measured with a Synergy 2 microplate reader (Biotek, Winooski, VT) to determine adherence. Wells containing mMRS with the corresponding sugar without *L. plantarum* inoculum were included as controls.

### Yeast inhibition assay

*Lactiplantibacillusplantarum* cell‐free culture supernatants (CFCS) were prepared from the spent media collected after *L. plantarum* incubation in cMRS for 24 h at 30°C. CFCS was collected by centrifugation at 4000× *g* for 10 min at 4°C followed by filtration of the supernatant through a 0.45 μm polyethersulfone (PES) filter (Genesee Scientific, San Diego, CA). To eliminate the effects of differences of pH on yeast inhibition, the CFCS was adjusted with lactic acid (1.3 M) to pH 3.8, the lowest pH reached by *L. plantarum* after incubation in cMRS (data not shown). *S. cerevisiae* UCDFST 09‐448 (Golomb *et al*., 2013), a strain shown to cause olive tissue damage and spoilage during olive fermentations, was grown in yeast mould (YM) broth (BD, Franklin Lakes, NJ) for 24 h at 30°C with aeration at 250 rpm. Cells were collected by centrifugation at 20 000× *g* for 5 min at 4°C and then washed twice with PBS. *S. cerevisiae* UCDFST 09‐448 was then inoculated into 96‐well microtiter plates containing 1:1 ratio of 2X YM and CFCS at a starting OD_600_ of 0.05. OD_600_ was measured in a Synergy 2 microplate reader (Biotek, Winooski, VT) set at 30°C for 24 h aerated every hour by shaking for 10 s before each read. Controls included *S. cerevisiae* UCDFST 09‐448 incubated in YM and YM supplemented with cMRS (pH 3.8).

### Statistical analysis

Area under the curve (AUC) was used to examine the growth and survival of *L. plantarum* under different conditions (Sprouffske and Wagner, 2016). The AUC was calculated with GraphPad Prism 8 (Graph Pad Software, San Diego, CA). Hierarchical clustering was generated using RStudio with the package ‘pheatmap’ based on AUC values (https://www.rstudio.com/). Unpaired, two‐tailed Student’s *t*‐tests were used to compare between the different *L*. *plantarum* groups (e.g. brine‐ and grain‐based fermentations). *P* values of < 0.05 were considered significant.

## Conflict of interest

The authors declare that the research was conducted in the absence of any commercial or financial relationships that could be construed as a potential conflict of interest.

## Supporting information

**Table S1.** Allelic profiles of L. plantarum strains according to MLST^a^.
**Table S2.** Average AUC values of L. plantarum under different growth conditions.
**Table S3.** Final OD600 of L. plantarum under different growth conditions.
**Table S4.** Growth rates of L. plantarum in mMRS containing different sugars.
**Table S5.** Growth rates of L. plantarum exposed to different environmental stresses.
**Table S6.** Growth characteristics of S. cerevisiae UCDFST 09‐448 incubated in L. plantarum cell free supernatant (CFCS).
**Table S7.** Distribution of gene clusters across *L. plantarum* genomes based on COG categories.
**Table S8.** PCR primers used in this study.
**Fig. S1.** Minimum spanning tree of L. plantarum using MLST.
**Fig. S2.** Growth of L. plantarum in mMRS containing 2% (w/v) raffinose after repeated incubation in that culture medium.
**Fig. S3.** Growth of L. plantarum in mMRS containing 2% (w/v) ribose after repeated‐incubation in that culture medium.
**Fig. S4.** Growth of L. plantarum in mMRS containing 2% (w/v) arabinose after repeated‐incubation in that culture medium.
**Fig. S5.** The core‐genome and pan‐genome of nine L. plantarum strains examined in this study.
